# A Narrative Review of Evolving Techniques in Nonsurgical Rhinoplasty: Risk Profile and Clinical Outcomes of Fillers

**DOI:** 10.7759/cureus.102303

**Published:** 2026-01-26

**Authors:** Sergio Camilo Torres Céspedes, Ahtziri Pagan, Ali Alotaibi, Giuseppe Antonio D Amico, Manar Almutiri, Gustavo Guillen, Bashir Imam, Amar Shamsah, Amaranta Manjarrez

**Affiliations:** 1 Medicine, Universidad Nacional de Colombia, Bogota, COL; 2 Medicine, University of Medicine and Health Sciences, Basseterre, KNA; 3 General Surgery, Al Adan Hospital, Ministry of Health, Al-Mubarak Al-Kabeer, KWT; 4 Plastic and Reconstructive Surgery, Azienda Ospedaliera Universitaria Policlinico "G. Martino", Messina, ITA; 5 Otolaryngology, Zain Hospital, Kuwait, KWT; 6 General Medicine, Caja Costarricense de Seguro Social, San Jose, CRI; 7 Pediatrics, University of Pittsburgh Medical Center, Coudersport, USA; 8 School of Medicine, Westhill University, Mexico City, MEX

**Keywords:** dermal fillers, hyaluronic acid, non-surgical rhinoplasty, patient satisfaction, ultrasound guidance, vascular occlusion

## Abstract

Nonsurgical rhinoplasty (NSR) is a minimally invasive alternative to surgical rhinoplasty that uses dermal fillers to enhance nasal aesthetics. When performed by trained injectors, NSR offers rapid correction with relatively low downtime. This narrative review explores the historical evolution, anatomical considerations, filler materials, patient selection, procedural techniques, risk mitigation strategies, and clinical outcomes associated with NSR. Hyaluronic acid (HA)-based fillers remain the gold standard due to their reversibility, biocompatibility, and favorable safety profile. Recent innovations, including ultrasound-guided injections, hybrid techniques combining HA with polydioxanone (PDO) threads, and AI-assisted facial mapping, have improved precision and patient satisfaction. Although most complications are transient, such as edema and erythema, rare but severe events like vascular occlusion, skin necrosis, and visual loss underline the importance of detailed anatomical knowledge and preventive techniques. The review also emphasizes individualized treatment approaches considering ethnic and anatomical variations. Overall, NSR demonstrates high efficacy, rapid recovery, and reversibility, with satisfaction rates exceeding 90% across multiple studies, reaffirming its position as a valuable aesthetic procedure when performed by trained professionals.

## Introduction and background

The history of nonsurgical rhinoplasty (NSR) dates back to the late 1800s, when the invention of the syringe made it possible to use injectable materials for facial aesthetics. This innovation enabled physicians to perform minimally invasive procedures [1]. Early practitioners began experimenting with chemical agents to restore facial contours, reconstruct deformities, and maintain a youthful appearance [1]. Pioneers such as Corning and Gersuny introduced liquid paraffin injections to correct saddle-nose deformities, one of the first recorded uses of fillers for aesthetic purposes. The technique’s ability to reshape facial contours and add volume through simple injection methods was quickly recognized by physicians [2,3].

Although initially hailed as a breakthrough, enthusiasm soon waned as delayed and severe complications such as embolization, granuloma formation, ulceration, and migration became evident [2,3]. These early efforts marked the first milestone in the evolution of modern liquid rhinoplasty, but the significant adverse outcomes associated with paraffin underscored the need for safer materials.

Subsequently, the medical community began emphasizing biocompatibility and safety in injectable substances. The development of bovine collagen injections in the 1980s represented a major advance in safety and efficacy compared to the permanent synthetic materials previously in use. This transition to biological materials laid the foundation for temporary fillers that would ultimately transform NSR [4].

The shift toward temporary and biocompatible fillers gained momentum as researchers addressed the risks and limitations of permanent injectables. Silicone-based fillers, which followed paraffin in the historical timeline, also caused serious complications [1]. Although silicone could achieve lasting results, it was later discouraged due to its association with severe granulomatous reactions in certain patients. Consequently, clinicians began to favor safer, biodegradable, and temporary fillers that could be naturally metabolized by the body [1].

The introduction of hyaluronic acid (HA) fillers marked a defining moment in the evolution of NSR. HA fillers dramatically improved both safety profiles and clinical outcomes [5]. Due to their low immunogenicity, reversibility, and ease of administration, HA fillers have become the most widely used injectable materials in facial aesthetics today [5]. Their safety and efficacy have enabled practitioners to achieve desirable cosmetic outcomes while maintaining patient well-being. Injectable HA hydrogels have grown increasingly popular because they offer durable results, minimal complications, no allergen testing requirement, and are easily reversible with hyaluronidase treatment [6]. This reversibility represented a major advancement, allowing patients to modify their results if desired.

Extensive research supports the safety, efficacy, and continued advancement of NSR techniques. The introduction of HA-based dermal fillers has made these procedures more accessible, with benefits including fewer side effects, faster recovery, and lower cost [7]. A recent meta-analysis published in 2024, involving 9,657 patients across multiple studies, reported that over 99% of participants were satisfied with their outcomes. Most complications were minor, such as transient swelling or redness, while only 0.27% experienced serious vascular events like skin necrosis, blindness, or stroke [8]. These findings reflect both the widespread adoption of the technique and the robust clinical data supporting its safety. When performed by trained professionals with sound anatomical knowledge, NSR is considered a safe and effective aesthetic procedure [9].

Modern NSR has evolved to include new materials and combination techniques. For instance, recent innovations have incorporated polydioxanone (PDO) threads alongside traditional fillers. The combined use of PDO threads and HA fillers has demonstrated safety and efficacy, with results lasting up to six months post-treatment. Threads are particularly useful for lifting the nasal tip and achieving better facial balance [10]. This multimodal approach represents the most advanced form of NSR, enabling physicians to address both volumetric enhancement and structural support through minimally invasive methods.

The transition from permanent synthetic materials to temporary biocompatible fillers reflects a deeper understanding of aesthetic principles, patient safety, and tissue response. This paradigm shift has transformed NSR from a high-risk experimental procedure into a well-established treatment with predictable results and manageable complications. The risk-benefit profile has improved significantly with the use of temporary fillers. HA-based dermal fillers are generally safe and effective, with most adverse effects being mild, transient, and self-limiting, while serious complications remain exceedingly rare [11].

Despite the growing popularity of NSR, the literature remains fragmented, with variations in reported techniques, filler materials, complication profiles, and outcome measures. Many existing reviews focus on individual filler types or isolated complications, without providing an integrated overview of evolving techniques alongside their associated risk profiles and clinical outcomes.

The objective of this narrative review is to synthesize current evidence on NSR, with particular emphasis on the evolution of injectable materials, procedural techniques, complication patterns, and clinical outcomes. This review aims to contextualize historical developments alongside contemporary practices, including combination approaches such as HA fillers with adjunctive modalities, to provide clinicians with a consolidated, clinically relevant overview. By integrating safety data, outcome trends, and procedural innovations, this manuscript offers an updated perspective on NSR that addresses recent advancements and emerging practices not comprehensively covered in prior reviews.

## Review

Anatomy of the nose relevant to NSR

A precise understanding of nasal vascular anatomy is fundamental to minimizing complications in NSR, as the majority of serious adverse events reported are vascular in origin [12,13]. Unlike surgical rhinoplasty (SR), injectable techniques rely on blind placement of filler within variable soft-tissue planes, increasing susceptibility to intravascular injection and embolic complications [14].

Burget and Menick's concept of nasal aesthetic subunits remains a cornerstone in nasal surgery and minimally invasive procedures, offering a practical framework for predicting scar camouflage, flap design, and complication distribution, rather than merely serving as a descriptive anatomical model [12]. The dorsum, tip, columella, paired alae, sidewalls, and soft triangles collectively form ten subunits that correspond with distinct vascular densities and clinical risk zones. The external nose receives its blood supply from branches of both the internal and external carotid arteries. Anatomical studies consistently demonstrate that the dorsal nasal, external nasal, lateral nasal, inferior alar, and columellar arteries form an interconnected subdermal vascular network rather than functioning as isolated vessels [13-16]. This anatomical configuration explains why dorsal injections, particularly along the radix and upper dorsum, are disproportionately associated with visual complications reported in filler-related adverse event registries [17].

Similarly, the lateral nasal and alar arteries, branches of the facial artery, supply the nasal sidewalls and tip. These vessels frequently course within or immediately superficial to commonly targeted injection planes, accounting for the higher incidence of skin ischemia and necrosis reported in the alar and tip regions [14,18]. The columellar artery, arising from the superior labial artery, further contributes to vascular density at the nasal base, reinforcing the need for heightened caution when augmenting tip projection or columellar support [19].

Clinical correlation between anatomy and complication patterns supports the use of strict midline injections and deep supraperiosteal placement along the dorsum, where major vessels are less commonly encountered [20,21]. Evidence from observational studies and complication analyses suggests that injections performed in deeper planes using low-pressure, small-volume techniques are associated with lower rates of vascular compromise, although risk cannot be eliminated [17,22]. Instead, contemporary injection strategies emphasize slow delivery, low-pressure techniques, the use of blunt cannulas where appropriate, and continuous needle motion to mitigate risk in these highly variable vascular territories [23]. Although multiple cadaveric studies describe nasal vascular anatomy in detail, most are limited by small sample sizes and lack correlation with real-world complication data. Existing evidence is therefore largely descriptive and inferential, highlighting the necessity for future studies integrating anatomical findings with clinical outcomes, imaging, and complication registries to define risk stratification in nasal procedures better.

Venous drainage largely parallels arterial anatomy and communicates with the angular vein and cavernous sinus. Although venous embolization is uncommon, this anatomical continuity has been proposed as a theoretical mechanism for intracranial spread following high-pressure injections [16,24].

**Figure 1:**
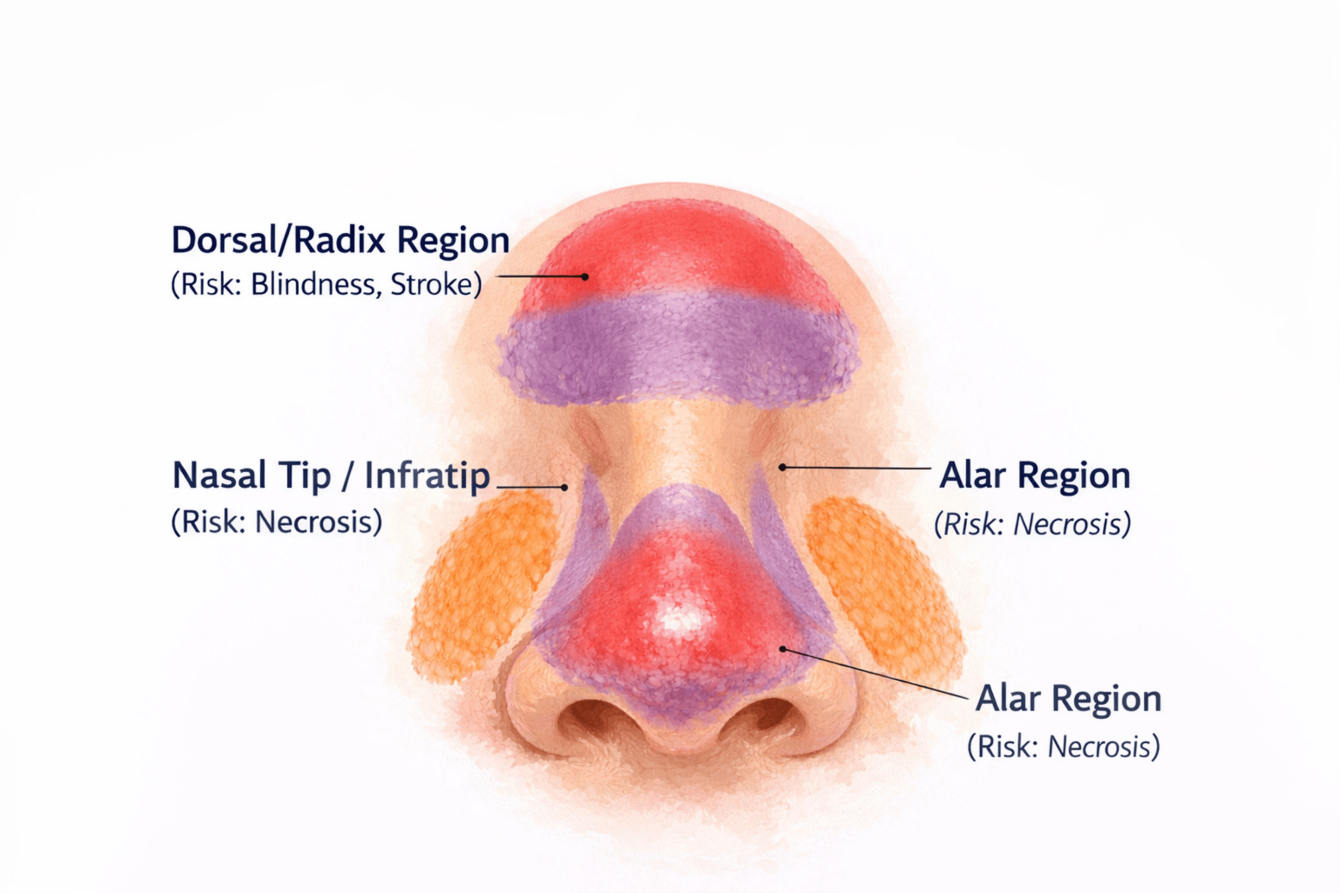
High-risk injection points on the nose. Image created using ChatGPT version 5.2 (OpenAI, San Francisco, CA).

Taken together, nasal vascular anatomy directly informs injection plane selection, product choice, and risk mitigation strategies. Rather than serving as a purely descriptive consideration, anatomical knowledge enables clinicians to anticipate complication patterns and tailor techniques accordingly, emphasizing the importance of anatomy-driven decision-making in NSR (Figure 1) [12,17,21].

Types of fillers used in NSR

Dermal fillers are widely used for facial rejuvenation and contour enhancement, offering minimally invasive alternatives to surgery. Historically, materials such as autologous fat and silicone oil were used, but modern practice relies on biocompatible and safer substances such as HA, calcium hydroxylapatite (CaHA), poly-L-lactic acid (PLLA), and polymethyl methacrylate (PMMA) [18-20]. Each filler type varies in composition, longevity, and mechanism of action, influencing its suitability for specific anatomical sites. In NSR, HA remains the filler of choice, and the only recommended one due to its reversibility with hyaluronidase and favorable safety profile [21-25]. Table 1 summarizes key characteristics of major filler types used in clinical practice.

**Table 1:** Summary of common dermal fillers used in aesthetic practice. Sources: [18-25].

Filler type	Composition	Mechanism/action	Duration of effect	Advantages	Limitations/considerations	Contraindications	Common brand names
Hyaluronic acid (HA)	Naturally occurring glycosaminoglycan	Volumizes tissue and hydrates the extracellular matrix	6-12 months	Reversible with hyaluronidase; safe and versatile	Risk of vascular occlusion; shorter duration	Previous nose implants, Rosacea, or vascular diseases	Juvederm, Restylane
Calcium hydroxylapatite (CaHA)	Calcium-based microspheres in gel	Stimulates collagen I & II formation and neovascularization	12-18 months	Longer-lasting, firm structure ideal for contouring	Not reversible; may cause nodules if injected superficially	Active skin infection, inflammation; Pregnancy; Immunosuppression	Radiesse
Poly-L-lactic acid (PLLA)	Biodegradable polymer	Induces fibroblast collagen synthesis	Up to 2 years (gradual onset)	Improves skin texture and firmness	Requires multiple sessions; delayed results	History of keloid or hypertrophic scar formation; Autoimmune disorders	Sculptra
Polymethyl methacrylate (PMMA)	Microspheres in bovine collagen gel	Semi-permanent structural filler	>5 to 10 years	Long-lasting volumization	Irreversible; risk of granuloma formation	Bleeding disorders; Previous fillers in the area; Active infections	Bellafill

Techniques and injection approaches

NSR relies on precise, anatomy-based techniques designed to enhance nasal contour while minimizing vascular risk. The procedure’s safety and outcome depend primarily on respecting the anatomical midline, employing low filler volumes, maintaining slow injection speed, and using aspiration and ultrasound guidance to prevent intravascular events [26-28].

In practice, injections are confined to the anatomical midline, avoiding lateral displacement where major vessels such as the dorsal nasal and lateral nasal arteries traverse. Filler is deposited in micro-boluses (<0.05 mL) at the radix, dorsum, and tip, typically within the deep supraperiosteal or perichondrial plane to minimize vascular compromise [27]. Aspiration before injection and slow, low-pressure delivery further reduce the likelihood of embolization or tissue ischemia [29,30].

A blunt-tip cannula (25-27 G) is preferred for dorsal augmentation due to its reduced risk of vascular penetration, while sharp needles may be used for targeted deep boluses in well-visualized midline areas [31]. The filler volume is individualized, and post-procedure massage is avoided to prevent displacement or vascular compression [26].

Emerging adjuncts such as ultrasound-guided injections enhance procedural safety by allowing real-time visualization of vascular anatomy, ensuring precise filler placement and early detection of potential complications [32-34]. Hybrid approaches, including the integration of HA fillers with polydioxanone (PDO) threads, have also been reported to improve tip support and definition, though their safety principles remain rooted in the same conservative, anatomy-conscious methodology [30].

Ultimately, successful NSR outcomes depend less on filler type and more on adherence to fundamental safety principles: strict midline placement, minimal injection volume, slow and controlled delivery, aspiration, and ultrasound confirmation of the vascular-free plane. These measures collectively minimize ischemic complications and optimize predictable, natural-appearing results [28-34].

Indications and patient selection

NSR is best suited for patients seeking subtle nasal enhancement through volume addition rather than structural reduction. Appropriate candidates typically present with minor contour irregularities, asymmetry, low radix, dorsal depression, or post-rhinoplasty surface defects and desire correction without surgical downtime [35-38]. HA fillers remain preferred due to their reversibility with hyaluronidase and established safety profile [39].

Patient selection should be guided by both aesthetic goals and medical suitability. Ideal candidates possess realistic expectations, healthy skin quality, and no significant anatomical deformities requiring structural correction. Conversely, individuals with severe deviations, large dorsal humps, or functional airway concerns should be referred for surgical evaluation [27,38,40-42].

Careful exclusion of medical and psychological contraindications is essential. Procedures should be deferred in pregnancy, active infection, or after recent nasal surgery or trauma. Autoimmune disorders, bleeding diatheses, or prior silicone or unknown filler use warrant caution. Screening for body dysmorphic disorder (BDD) is mandatory, as affected patients often demonstrate poor satisfaction and adverse psychological outcomes despite technically adequate results [43-45].

Comprehensive screening for systemic illness, prior filler history, and psychological suitability remains fundamental to safe NSR practice. Excluding patients with BDD or medical contraindications reduces the risk of complications and ensures ethical, predictable outcomes Table 2 [35-45].

**Table 2:** Summary of patient selection criteria for nonsurgical rhinoplasty. Sources: [35-45].

Category	Inclusion criteria (suitable candidates)	Exclusion/contraindications
Anatomical/Aesthetic	Mild dorsal depression or radix deficiency; tip ptosis, minor asymmetry, post-rhinoplasty irregularities; desire for augmentation rather than reduction	Severe dorsal hump or deviation, functional airway obstruction, damaged skin, and recent nasal surgery or trauma
Medical	Healthy, immunocompetent patient; no active infection or inflammation	Pregnancy or breastfeeding; uncontrolled diabetes or bleeding disorders; autoimmune disease; active skin infection, dermatitis, or radiotherapy to the nose
Treatment history	No previous filler or known HA-based injections and adequate healing time after prior cosmetic procedures	Prior use of silicone or unknown permanent fillers; recent laser, chemical peel, or invasive skin procedures
Psychological	Realistic expectations and stable body image; informed understanding of the temporary nature of results	Body dysmorphic disorder (BDD) or unrealistic aesthetic demands; unstable psychiatric conditions
Medication/Systemic	Off anticoagulants, where clinically safe; no immunosuppressive therapy	Ongoing anticoagulant or corticosteroid use (unless optimized)

Risk profile and complications 

Although NSR using dermal fillers is generally safe, complications may range from mild and transient to severe and vision-threatening. Understanding their presentation, mechanisms, and frequency is essential for effective prevention and management. Recent systematic reviews report overall complication rates below 1%, with vascular occlusion (0.35%), visual loss (0.09%), skin necrosis (0.08%), and infection (0.07%) representing the most serious but rare events [42-50].

Common transient events

The majority of post-procedural effects are mild, short-lived, and self-limiting. These include erythema, edema, tenderness, and ecchymosis, typically resolving within 7-10 days [46]. Superficial filler placement may occasionally produce the Tyndall effect, leading to bluish discoloration; this can be reversed with hyaluronidase [47]. Proper midline injection, low filler volume, and avoidance of superficial planes substantially reduce these risks.

Infectious and granulomatous complications

Bacterial infections (e.g., Staphylococcus aureus) and biofilm formation are uncommon but clinically significant. They may present as delayed induration, erythema, or abscess formation requiring antibiotics or, rarely, drainage [47-53]. Granulomatous and delayed hypersensitivity reactions occur in <0.5% of cases, typically associated with semi-permanent fillers or prior biofilm activation [47,51]. Reactivation of herpes simplex or local cellulitis can occur in predisposed individuals but remains rare. Preventive measures include strict asepsis, appropriate filler depth, and avoidance of treatment during active dermatoses.

Vascular emergencies

Vascular occlusion is the most serious complication of NSR, accounting for approximately 80% of all filler-related ischemic events [47]. It may result from intravascular injection or external compression of key arteries, particularly the dorsal nasal and angular branches, which communicate with the ophthalmic system [54-58]. Early symptoms include pain, pallor, and livedo progressing to necrosis or visual loss if untreated. Reported incidence is 0.35% for occlusion and 0.09% for blindness [42]. Immediate management includes high-dose hyaluronidase, warm compresses, vasodilators, and, when indicated, urgent ophthalmologic referral for potential intra-arterial thrombolysis [56].

Cutaneous necrosis, most often involving the nasal tip, occurs in approximately 0.08% of cases and typically within hours of injection [42]. Prevention relies on slow, low-pressure injection, midline placement, and ultrasound guidance to avoid vascular structures.

Strategies for risk reduction of filler-related complications

Risk mitigation in NSR depends on anatomical precision, safe injection technique, and early recognition of ischemic signs. While complications cannot be eliminated entirely, adherence to standardized safety principles markedly reduces their likelihood [46,47,55].

Anatomical and technical principles

Injections should remain strictly midline and in the deep supraperiosteal or perichondrial plane, avoiding the lateral vascular zones where the dorsal nasal and angular arteries course [55]. Low-volume, slow, retrograde injection with intermittent aspiration reduces intravascular entry risk. Blunt-tip cannulas (25-27 G) or fine needles (27-30 G) may be used, depending on operator expertise and target depth, but should never be advanced forcefully against resistance.

Use of ultrasound and visualization

High-frequency Doppler ultrasound allows real-time mapping of nasal vasculature, confirmation of injection depth, and identification of low-flow “safe zones.” Evidence shows it significantly reduces the incidence of vascular occlusion and blindness when incorporated into practice [57,59]. Its use is recommended particularly for revision cases or high-risk zones such as the radix and glabella.

Patient and procedural screening

A detailed medical history, including vascular disease, coagulopathies, prior fillers, and autoimmune disorders, helps identify high-risk individuals. Procedures should be deferred in patients with recent nasal trauma, infection, or pregnancy. Informed consent and documentation of pre-procedure visual function form part of good clinical practice [47,60].

Early detection and response

Immediate blanching, disproportionate pain, or resistance to injection should prompt cessation of filler delivery and rapid administration of hyaluronidase, warm compresses, and massage to restore perfusion. Early ophthalmologic consultation is critical if visual symptoms arise. Prevention remains the most effective management strategy [55,57].

Management of filler-related complications and patient satisfaction

The management of filler-related complications in NSR aims to restore perfusion, prevent necrosis, and address both inflammatory and vascular events rapidly. While most adverse effects are mild and self-limiting, vascular compromise represents the most urgent scenario requiring prompt, evidence-based intervention [46,47].

General and Early Management

Transient edema, erythema, and bruising are managed conservatively with cold compresses, head elevation, and avoidance of exertion. The Tyndall effect from superficial filler placement responds well to massage and hyaluronidase injection [47].

For inflammatory nodules or granulomas, short courses of intralesional corticosteroids or antibiotics are preferred, while surgical drainage is reserved for refractory cases. Local infections are treated with beta-lactam or cephalosporin antibiotics, and patients should be monitored for delayed hypersensitivity reactions [47,61].

Vascular Occlusion and Ischemia

Immediate recognition of pain, pallor, or livedo is vital. The first-line therapy is high-dose, pulsed hyaluronidase, ideally within 4 hours of symptom onset. According to meta-analytic data, hyaluronidase use yields partial or full recovery in ~84% of vascular occlusion cases, especially when administered within 1-5 days [57]. Adjunctive measures include warm compresses, gentle massage, oral aspirin, topical nitroglycerin, and systemic corticosteroids to reduce inflammation and promote reperfusion [55,57].

Hyperbaric oxygen therapy (HBOT) or vasodilators may be considered as supportive interventions in persistent ischemia, although evidence remains limited [62].

Ocular or Visual Compromise

Ocular vascular occlusion requires urgent multidisciplinary management. The goal is to reduce intraocular pressure and restore retinal perfusion. Reported interventions include retrobulbar hyaluronidase injection, anterior chamber paracentesis, ocular massage, and systemic corticosteroids. Timely initiation, preferably within 90 minutes, is critical to visual recovery [49,56]. Despite varied protocols, the consensus emphasizes early recognition and rapid referral to ophthalmology for targeted reperfusion therapy.

Role of Hyaluronidase

Hyaluronidase remains the cornerstone of managing HA-related complications. Beyond dissolving filler material, it exerts anti-edematous and microcirculatory benefits that facilitate tissue reperfusion [47]. The DeLorenzi high-dose pulsed hyaluronidase (HDPH) protocol is now considered the standard of care, involving repeated high-dose administration until clinical improvement [63]. Ultrasound-guided injections enable direct visualization of affected vessels, optimizing outcomes while avoiding unnecessary tissue injury [48,64]. Although rare, clinicians must remain vigilant for anaphylactic reactions following enzyme administration [65].

**Table 3:** Summary of management strategies for filler-related complications. Sources: [46-49, 55-57, 61,62,64]. HBOT, hyperbaric oxygen therapy

Complication type	Primary intervention	Adjunctive/supportive measures	Evidence/outcome
Mild edema/bruising	Cold compresses, rest	Avoid heat, exercise, and anticoagulants	Self-limiting (resolves in 3–7 days)
Tyndall effect	Hyaluronidase (localized)	Gentle massage	Complete correction expected
Inflammatory nodule/granuloma	Intralesional corticosteroids, antibiotics	Drainage if refractory	Resolution in the majority within weeks
Vascular occlusion/necrosis	High-dose pulsed hyaluronidase within 4 hours	Aspirin, topical nitroglycerin, warm compress, corticosteroids, HBOT	~84% recovery with early treatment
Ocular ischemia/blindness	Retrobulbar hyaluronidase, paracentesis, ocular massage, ophthalmology referral	IV mannitol, corticosteroids, oxygen therapy	Vision recovery variable; prognosis time-dependent

Clinical outcomes and patient satisfaction

Meta-analyses indicate that NSR achieves 80%-100% immediate patient satisfaction, with sustained rates above 80% at 12 months, and overall complication rates below 1% [66]. Compared with SR, NSR offers lower morbidity, faster recovery, and reversibility. While SR yields higher long-term structural refinement, it carries higher revision and complication risks, including septal perforation and persistent nasal obstruction [66]. Hence, NSR represents an effective, low-risk aesthetic intervention when performed by trained injectors with strong anatomical understanding and early recognition of complications [66].

Limitations

This review is narrative in nature and does not follow a systematic review methodology. As a result, study selection may be subject to selection bias, variability in evidence quality, and heterogeneity in reported outcomes. The conclusions presented are intended to provide an informed overview of evolving techniques, risk profiles, and clinical trends in NSR rather than definitive or generalizable evidence. Nevertheless, narrative reviews remain valuable for contextualizing historical evolution, emerging practices, and real-world clinical considerations, particularly in rapidly evolving aesthetic fields.

Advances and future trends

Recent innovations have enhanced the safety, precision, and personalization of NSR. Doppler ultrasound now serves as a key tool for visualizing nasal vasculature in real time, enabling injectors to identify safe planes, avoid critical arteries, and adjust needle position intra-procedurally, significantly reducing the risk of intravascular injection and blindness [59,67].

The integration of artificial intelligence (AI) has transformed facial analysis and treatment planning. Using 3D modeling and augmented-reality systems (e.g., ModiFace, Crisalix), AI allows objective assessment of facial symmetry, prediction of outcomes, and personalized procedural design. These systems improve patient communication and expectation management but raise ongoing concerns regarding data privacy and algorithmic bias [68,69].

Technological advances in filler formulation, such as Vycross cross-linking and biostimulatory materials like CaHA and PLLA, offer longer-lasting effects (up to two years) by enhancing collagen stimulation and structural support [70,71].

A growing trend involves multimodal rejuvenation, combining threads, fillers, and energy-based devices (radiofrequency or ultrasound) to achieve lift, volume restoration, and collagen remodeling in one protocol. This synergy enhances contour refinement and durability when tailored to individual anatomy and aesthetic goals [54].

Collectively, these advances reflect a shift toward evidence-guided, technology-assisted, and patient-specific approaches that improve both procedural safety and satisfaction in NSR. Integrating standardized outcome measures with long-term follow-up in future studies will be key to building a stronger evidence base. This, in turn, will help inform consensus-based guidelines and improve personalization of NSR treatments.

## Conclusions

NSR represents a paradigm shift in aesthetic facial enhancement, prioritizing safety, reversibility, and patient satisfaction over invasive surgical correction. With advances in dermal filler technology, ultrasound guidance, and hybrid procedural techniques, NSR has achieved a strong balance between predictable outcomes and reduced complication rates. However, its success is contingent upon meticulous patient selection, comprehensive anatomical understanding, and skilled injector technique. HA fillers remain the cornerstone of practice due to their safety and reversibility, while emerging technologies, such as AI-driven facial analysis and bio-stimulatory fillers, promise further refinement. As patient demand for minimally invasive procedures grows, standardized training and evidence-based protocols will be vital to optimize safety and sustain long-term satisfaction.
